# Ecological associations distribution modelling of marine plankton at a global scale

**DOI:** 10.1098/rstb.2023.0169

**Published:** 2024-07-22

**Authors:** Marinna Gaudin, Damien Eveillard, Samuel Chaffron

**Affiliations:** ^1^ Nantes Université, École Centrale Nantes, CNRS, LS2N, UMR 6004, Nantes 44000, France; ^2^ Research Federation for the Study of Global Ocean Systems Ecology and Evolution, FR2022/Tara Oceans GOSEE, Paris 75016, France

**Keywords:** ecological associations, species distribution modelling, statistical learning, marine plankton, metagenomics, climate change

## Abstract

Marine plankton communities form intricate networks of interacting organisms at the base of the food chain, and play a central role in regulating ocean biogeochemical cycles and climate. However, predicting plankton community shifts in response to climate change remains challenging. While species distribution models are valuable tools for predicting changes in species biogeography under climate change scenarios, they generally overlook the key role of biotic interactions, which can significantly shape ecological processes and ecosystem responses. Here, we introduce a novel statistical framework, association distribution modelling (ADM), designed to model and predict ecological associations distribution in space and time. Applied on a *Tara* Oceans genome-resolved metagenomics dataset, the present-day biogeography of ADM-inferred marine plankton associations revealed four major biogeographic biomes organized along a latitudinal gradient. We predicted the evolution of these biome-specific communities in response to a climate change scenario, highlighting differential responses to environmental change. Finally, we explored the functional potential of impacted plankton communities, focusing on carbon fixation, outlining the predicted evolution of its geographical distribution and implications for ecosystem function.

This article is part of the theme issue ‘Connected interactions: enriching food web research by spatial and social interactions’.

## 1. Introduction

Marine plankton form highly complex and dynamic communities that play essential roles in the biosphere, underpinning ocean productivity [1,2], contributing to the regulation of biogeochemical cycles, sustaining half of the world’s primary production [2], and constituting the base of the global marine food web [3]. Planktonic communities are mainly influenced by their physico-chemical context, which shapes their metabolism, physiology, phenotype and, at a broader scale, their biogeographical distributions [4]. Consequently, ongoing climate change poses a severe threat to the balance of these communities [5]. Recent reports have documented significant alterations in plankton community distributions [6,7], highlighting the pressing need to understand and predict the nature and amplitude of these responses to environmental disturbances. A classical approach for assessing a community structure focuses on its elements and their projection into biogeographies [8], which has led to the development of species distribution modelling (SDM) [9]. SDM aims to relate observed species locations and associated physico-chemical variables, typically using statistical methods to predict and describe species biogeographies in space and/or time [10]. The resulting models (SDMs) can subsequently characterize each cell of a map by the suitability of species occurrence as a function of the environmental conditions present in that cell. SDMs have been extensively used to forecast how planktonic species might respond to climate change scenarios. Most of these studies converge on the idea that significant poleward shifts in species distribution are likely to occur [11–13] owing to changes in temperature, salinity and nutrient concentration, which have been identified as the main factors shaping the distribution of planktonic organisms [14]. Such changes are expected to significantly impact the dynamics of associated ecosystem functions, including primary production, carbon fixation and nitrogen cycling [14–17].

While previous efforts to protect biodiversity have mainly centred around individual species, recent works suggested focusing on community interactions [18,19]. Marine plankton species are interconnected through a complex network of biotic interactions, including competition, predation, mutualism and parasitism, which involve exchanges of material, energy and information [20]. While SDMs can offer valuable insights into the biogeography and evolution of planktonic ecosystems, they do not capture the role of biotic interactions in structuring plankton communities [21]. Several studies have shown that biotic interactions are crucial in shaping community structure, dynamics and distributions [22]. A comprehensive review of one hundred studies [23] has revealed significant variability in both the strength and direction of impacts of global factors on diverse biotic interactions, highlighting their sensitivities to abiotic factors and their specific biogeographical patterns [24]. In addition, it has been documented that species loss and interaction loss occur at different rates [19], with interactions often being lost at a higher rate, especially for highly specialized and rare interactions, outlining an increased vulnerability [25].

The dynamic nature of biotic interactions and their non-stationarity regimes have generated considerable interest in species distribution modelling. Consequently, numerous studies have attempted to integrate indices of biotic dynamics into SDMs, mainly using three approaches [26]: (i) the integration of pairwise dependencies [27,28], (ii) the use of integrative predictors [29,30], and (iii) the hybridization of SDMs with dynamic models [31,32]. Overall, these works have improved the predictive performance of species distributions [21], reinforcing the fact that biotic interactions play an essential role in shaping species distributions and may strongly influence how climate change affects planktonic community responses at various scales [26]. Consequently, community-level modelling (CLM) frameworks [33] were designed with the aim to model community distributions as a whole, while offering deeper insights into biotic interactions. Notably, the development of joint species distribution models (jSDMs) has provided more insights into biotic interactions by simultaneously modelling joint distributions of multiple species [34]. However, they usually fail in capturing the spatial variability of biotic interactions, as they provide single and static correlation scores obtained from model residuals [35,36]. Detecting direct and indirect biotic interactions embodies a substantial empirical challenge, even in relatively simple ecological systems [37].

The advent of large-scale sampling, high-throughput sequencing and resulting omics data have allowed the application of statistical (learning) methods to advance our holistic understanding of plankton community dynamics [38]. In particular, co-occurrence analyses have been widely used to infer ecological associations under the assumption that interacting species display non-random co-occurrence patterns, indicating potential dependencies or interactions between species [39]. This has led to the use of co-occurrence graphs, integrating significant ecological associations, as valuable models for enhancing our comprehension of species community structure, stability and dynamics [28,40,41]. However, co-occurrence analyses usually consider statistical associations as static entities, not considering the spatiotemporal variability of biotic interactions [42]. To address this gap, we designed and developed a statistical framework called association distribution modelling (ADM), which enables the prediction of spatiotemporal distributions of planktonic ecological associations by modelling the statistical decomposition of their co-occurrence and co-abundance distributions. To illustrate the applicability of the ADM framework, we leveraged *Tara* Oceans genomic datasets, identifying thousands of significant ecological associations self-organized in biome-specific plankton communities along a latitudinal gradient. Based on a climatic scenario, we unravel diverse potential responses to climate change by predicting the spatiotemporal evolution of these ecological communities until the end of this century. Our findings shed light on community network structure responses to environmental change, providing valuable information about complex interactions within planktonic communities, and their dynamic adaptation to changing environmental conditions.

## 2. Results

### (a) Association distribution modelling uncovers ecological associations biogeography

**Figure 1.**
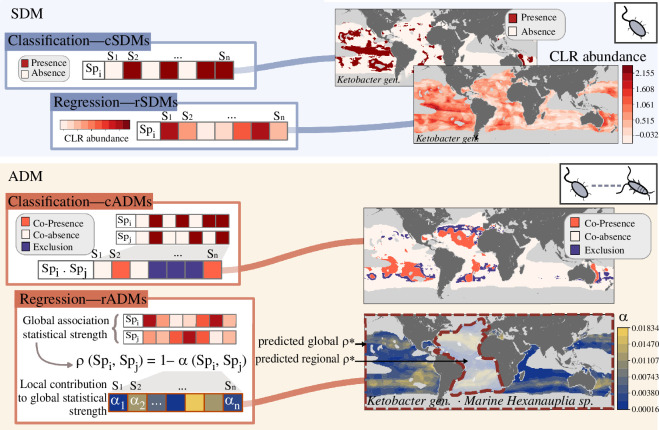
Schematic overview of the ADM conceptual framework in parallel with the SDM approach. The starting point involves observed counts (or abundances) of m species (or metagenomics assembled genomes) across n samples. After imputting data from the World Ocean Atlas18 climatologies as samples, species biogeography using SDMs or ecological associations between species using ADMs can be modelled and projected in space and time through WOA18 [46] or IPSL [47] climate model data. cSDMs predict species presence and absence, while rSDMs predict species abundance. Here, cADMs project species co-presence, co-absence and exclusion, revealing potential species interaction biogeographies, while rADMs estimate local statistical association strengths α, derived from decomposing the global statistical association strength ρ. These estimates enable the inference of predicted statistical forces $\rho^{*}$ at global or regional scales, as exemplified in the Atlantic Ocean region (lower right map). CLR, centred log ratio.

SDM allows the study of species distributions in space and/or time by relating the prevalence or abundance of species as a function of associated physico-chemical variables usually employing statistical methods. Individual SDMs can be inferred for each species as a statistical model approximating the species environmental niche. These models can be applied to other datasets with matching physico-chemical conditions, whether observed, simulated or modelled, enabling the prediction and analysis of species distributions. In this study, we refer to cSDM (classification-based SDM) and rSDM (regression-based SDM), which are respectively used to predict the occurrence and abundance of species distributions in each map grid cell. However, the question of whether an SDM model can generate reliable predictions of species abundance remains open [43], and one may refer to density surface models (DSMs) to improve the reliability of abundance predictions [44,45]. Here, we introduce ADM, a novel statistical framework designed to model and predict the distributions of ecological associations, that is, species pairs co-occurrence or co-abundance. ADMs model ecological associations as statistical proxies for potential biotic interactions to integrate their role in shaping biogeographical patterns (figure 1). We developed two variants of ADMs: cADMs (classification-based ADMs) and rADMs (regression-based ADMs), which are used to model the co-occurrence (i.e. from discrete counts such as presence/absence) and co-abundance (i.e. from abundance) of species pairs, respectively. For a given species pair (A,B), an association can be discretized into co-occurrence information across samples, where co-presence, co-absence and exclusion are the three possible states. A cADM will seek to model an observed ecological association co-occurrence as a function of physico-chemical predictors using multiple regression approaches (e.g. random forest, gradient boosting tree) and project it in space and time. When quantitative and semi-quantitative abundance data are available, rADM will seek a similar objective but integrating abundance information (i.e. co-abundance).

To demonstrate the utility of the ADM framework, which can be applied to both discrete and continuous (including compositional) data, we built rADMs using metagenomics data from the *Tara* Oceans project [48]. Metagenomics data correspond to species relative abundance in each sample, resulting in what is known as compositional data [49]. Thus, classical correlation metrics applied to these data can lead to inferring erroneous associations [50]. To properly detect associations from compositional data, a proportionality metric, denoted as ρ, was introduced in Quinn *et al.* [51] as a valid metric as it accounts for the logarithmic relationship between species relative counts [52,53] (§4, equation (4.1)). ρ quantifies the global strength of associations and ranges between −1 and 1 to reinforce its analogy with correlation. Briefly, ρ represents the sum of normalized local deviations of species abundances involved in the association (see §4b,c for details). The ADM framework (here rADM) decomposes the ρ formulation into α, which represents the vector of local contributions to the global statistical strength ρ (cf. §4c, equations (4.3)–(4.7)). To infer an rADM, the observed α is modelled as a function of physico-chemical predictors using multiple regression approaches (e.g., random forest). $\alpha^{*}$ is the predicted vector α from the model. For a pair of species (A,B), a single $\alpha (A_{i},B_{i})$ reflects the local contribution or deviation from the mean of abundances at sample i, normalized by the sum of their variances. A single $\alpha (A_{i},B_{i})$ does not hold ecological significance, but by summing predicted $\alpha^{*}$, we can infer a projected global association strength for a given species pair, denoted $\rho^{*}$, or by summing a subset of $\alpha^{*}$ for a specific region (electronic supplementary materials, S1 and S2). A set of low values in α corresponds to a ρ≈1, indicating high co-abundance between species. In contrast, a set of high values in α results in ρ≈−1, denoting low co-abundance between the modelled species pair. To assess the ecological significance of α, we calculated correlations between observed α distributions and WOA18 environmental variables, which were then compared with correlations obtained with randomly sampled α distributions. α values appeared to be primarily driven by temperature and, to some extent, by silicates when compared with randomly permuted α (electronic supplementary material, figure S1). In contrast, relative and CLR abundances of species appeared to be influenced by temperature and salinity, with no significant pattern emerging for nutrients. These results suggest that α values hold proper ecological significance diverging from species abundances, thus supporting the assumption that ecological associations display distinct spatiotemporal distributions.

To further our understanding of marine plankton biogeography and ecosystem functioning, we applied both ADM variants (cADM and rADM) on *Tara* Oceans metagenomics data. We leveraged 2601 metagenomics assembled genomes (MAGs), including 1888 prokaryotic [54] and 713 eukaryotic MAGs [55], from three size fractions (i.e. 0.22–1.6/3, 20–180 and 0.8−5/2000 μm), for 80 samples whose distribution is depicted in electronic supplementary material, figure S2 (§4a). ADMs were then built using matching environmental variables using the World Ocean Atlas 18 (WOA18), which provides available climatological data on 1° longitude/latitude grids [46] (§4d(i)). For building ADMs, we tested and compared several learning algorithms of which the random forest technique systematically demonstrated better prediction performances than gradient tree boosting and support vector machine for both cADMs and rADMs (electronic supplementary material, S3 and figure S3 [56]). Consequently, we used projections generated through random forest for subsequent analyses.

### (b) Revealing present-day marine plankton ecological associations biogeography

Next, to reveal and analyse the global biogeographical distribution of present-day marine plankton ecological associations, we selected 17 518 ADMs of 103 440 computed models, focusing on positive association models yielding superior predictive performance (electronic supplementary materials, S3.2 and S4). We then used non-supervised clustering on respective cADMs (§4d(iii)) projections to identify and characterize major clusters or communities of species associations sharing biogeographical signatures. To avoid introducing significant uncertainties through extrapolations, we constrained our predictions from extending beyond the observed feature ranges [57]. Thus, the projections were limited to grids where the physico-chemical variables derived from WOA18 were between quantiles 2.5 and 97.5 of the corresponding variables in the *Tara* Oceans samples (see §4d(i); electronic supplementary material, S5).

**Figure 2.**
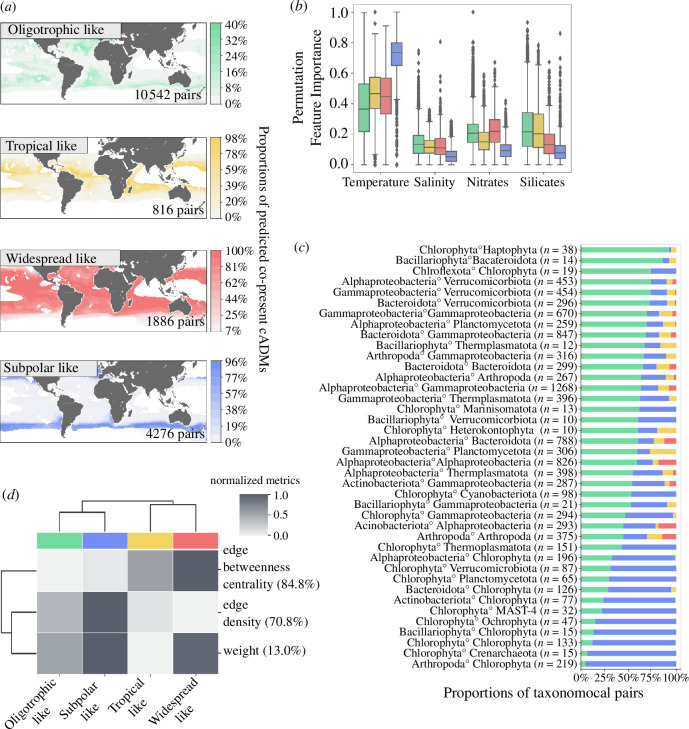
Main co-occurrence distribution signatures of positive ecological associations. (*a*) Maps depicting the distribution of associations co-presence proportions in grid cells for each identified cluster. (*b*) PFI highlights the drivers of distribution patterns. (*c*) Taxonomic composition of positively associated taxa within each cluster. The top 40 taxa association pairs and associations involving Chlorophyta, Bacillariophyta and Dinoflagellata are depicted. (*d*) Normalized graph topological metrics capturing edge-related features in inferred graphs of ecological associations within each cluster. The percentage in parentheses represents the difference proportion between the true (i.e. not normalized between 0 and 1) maximal value and the true minimal value.

These signatures were identified based on cADM projections at each geographical grid at present-day using WOA18 climatologies (§4d(iii)). Clustering these signatures identified four main distinct spatial patterns of ecological associations (figure 2*a*; electronic supplementary material, figure S4). The identified biogeographical groups were characterized based on their physico-chemical variables (electronic supplementary material, figure S5): (i) an oligotrophic-like cluster characterized by lower nutrient concentrations at temperate latitudes (green, 10 542 associations), (ii) a tropical-like cluster marked by higher temperatures (yellow, 1884 associations), (iii) a widespread-like cluster observed across all regions without any prominent distinct climatological characteristics (red, 816 associations) and (iv) a subpolar-like cluster characterized by colder temperatures and higher nutrient concentrations (blue, 4276 associations). The spatial distributions of these clusters underscored a structuration of projected ecological associations along a latitudinal gradient extending from subpolar to tropical latitudes. These four clusters reflected distinct communities with variable proportions of associated taxonomic pairs (figure 2*c*; electronic supplementary material, figures S6 and S7), underpinning unique biogeographically dependent ecological signatures; thus, supporting the sensitivity of ADMs for predicting biotic interactions under environmental forcing.

We predicted co-presences for all taxa (at phylum or class levels) detected within the oligotrophic-like and subpolar-like groups, albeit to large varying degrees (figure 2*c*). Notably, taxonomic pairs identified in the widespread-like clusters are also present in other clusters, reflecting their ubiquitous co-occurrence. For instance, alpha- and gamma-proteobacteria co-occurrences align with their well-documented prevalence in diverse environments [58,59]. A higher diversity in taxa associations characterizes the subpolar-like cluster compared with other clusters (electronic supplementary material, figures S6 and S7), as well as an overall higher predicted global $\rho^{*}$ (electronic supplementary material, figure S8*a*), in concordance with a previously reported increase in connectivity between species in polar areas [40]. Interestingly, taxa associations between Bacillariophyta (diatoms) and prokaryotes (Bacteroidota, Verrucomicrobiota and Gammaproteobacteria) are mainly attributed to the oligotrophic-like cluster. On the other hand, Bacillariophyta associations with eukaryotes (here Chlorophyta) are predominantly found in the subpolar cluster, potentially suggesting competitive relationships in nutrient-rich waters [60]. Moreover, subpolar-like communities harboured a higher proportion of Euk–Prok associations, while oligotrophic-like communities displayed a higher proportion of Prok–Prok associations (electronic supplementary material, figure S9), likely highlighting differential self-organization processes driving the assembly of heterotrophic microbiomes associated with autotrophic communities (e.g. mainly diatoms in subpolar ecosystems and mainly cyanobacteria in oligotrophic ecosystems). This is in line with the observation that unproductive regions (i.e. oligotrophic) are usually characterized by very high relative heterotrophic biomasses, while productive areas (i.e. subpolar) are usually characterized by a smaller contribution of heterotrophs to community biomass [61]. Overall, the exploration of these distinct community clusters reveals the dynamic nature of ecological associations that can thus be directly impacted by environmental forcing. Notably, ADMs can also inform community ecology at a more local (e.g. regional) scale by reconstructing predicted $\rho^{*}$ by ocean provinces, illustrating the local variability captured (electronic supplementary material, figure S8*b*). Despite the (expected) trend of stronger global $\rho^{*}$ in the subpolar (electronic supplementary material, figure S8*a*), and also in the North Atlantic Ocean, we observed higher regional $\rho^{*}$ for the oligotrophic cluster in globally more oligotrophic regions (South Pacific Ocean, South Atlantic Ocean and Indian Ocean), illustrating the consistency and usefulness of regional $\rho^{*}$ reconstruction. Also, ADM projections allow us to explore the biogeography of ecological associations involving a single species of interest, breaking down its distribution patterns for potential interaction partners in a given environmental context.

Given the significant influence of the environmental context in structuring ecological associations, we sought to identify the relative importance of environmental factors in shaping predicted community structures. The Permutation Feature Importance (PFI) index [62] offers a means to estimate the contribution of different features in shaping predicted co-occurrence distributions, providing insights into key drivers of ecological associations distributions (electronic supplementary material, S6) [63]. Here, we used PFI to gain insights into the relative importance of temperature, salinity, nitrates and silicates in shaping the predicted biogeographical co-occurrence patterns (figure 2*b*, §4d(i) for features selection). Temperature, often recognized as the most prominent driver of species biogeography [14,64,65], was identified as the main factor structuring co-occurrence biogeography, with the subpolar cluster being particularly temperature sensitive. The oligotrophic-like cluster had a stronger predicted sensitivity to nutrient concentrations, specifically nitrates and silicates. Similarly, this aligns with the lower nutrient concentrations observed in these areas, hence increasing the relative importance of nutrients in accurately predicting the spatial distributions of these cluster associations. Interestingly, the widespread-like cluster displayed a similar sensitivity to nitrates, potentially owing to its high number of taxa associations involving Alphaproteobacteria (i.e. Pelagibacterales (SAR11), Rhodobacterales and Puniceispirillales (SAR116)), which are usually capable of nitrate reduction [66,67]. The tropical-like cluster did not show a clear dominant driver, suggesting a combined influence of multiple parameters, including temperature and nutrient concentration.

Considering the environmental context significantly shapes predicted ecological associations, the reconstructed graphs topological properties can unravel emergent ecological network properties within each community cluster (figure 2*d*). Graph formal abstractions describe these networks, enabling the analysis of their structural and functional characteristics [40,68]. We reconstructed graphs for the four communities and computed topological metrics with the goal to uncover biome-specific topological features characterizing the structure and dynamics of ecological associations. We focused on topological metrics integrating information of graph edges, here representing ecological associations between species. To evaluate the ability of associations in connecting different parts of the graph, edge betweenness can serve as a proxy for information spread within networks [69], supporting resource and information flow between species. Edge betweenness centrality is calculated as the sum of the fraction of the shortest paths of all the pairs that pass through a particular edge (§4d(iii)). The widespread-like cluster showed a particularly high edge betweenness, suggesting key taxa associations across ocean regions, facilitating resource and interaction transfer among communities. The subpolar cluster stands out with a highly interconnected graph structure, emphasized by a more significant edge density (i.e. the ratio of the number of edges over the number of nodes) and high association weights ($\rho^{*}$). This suggests a greater level of connectivity among species, as well as more robust and consistent predicted biotic associations between them. This pattern was previously observed in a global plankton interactome [40] and may arise from higher species turnover and environmental variability in subpolar biomes, likely leading to denser and more dependent biotic interactions among distinct species. This result is to be considered in parallel to lower betweenness topological metrics. Average betweenness is expected to decrease with higher edge density or connections between nodes owing to the creation of multiple alternative paths [70]. From an ecological perspective, there may be a higher probability of direct interactions between species within the subpolar cluster, resulting in a more effective and direct exchange of nutrients and resources among the associated organisms in this cluster [71].

### (c) Evolution of ecological associations under a climate change scenario

**Figure 3.**
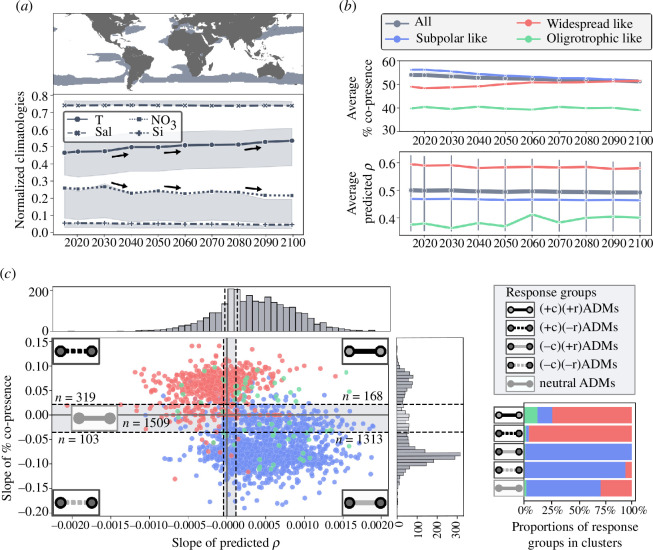
Deciphering plankton community spatial and temporal evolution in response to climate change. (*a*) Covered projectable area of IPSL RCP4.5 climate model and evolution of physico-chemical variables over 10 time points (2015, 2020 to 2100 with a 10-year step). (*b*) Evolution of co-occurrence percentage and predicted global $\rho^{*}$ across clusters over time. (*c*) Determination of five response groups of ADMs based on slopes encapsulating either the increase or decrease of co-occurrence proportion or $\rho^{*}$ over time: (+c)(+r)ADMs with increasing co-occurrence proportion and $\rho^{*}$ over the century, (+c)(−r)ADMs with increasing co-occurrence proportion but decreasing $\rho^{*}$ over the century, (−c)(+r)ADMs with decreasing co-occurrence proportion but increasing $\rho^{*}$ over the century, (−c)(−r)ADMs with decreasing co-occurrence proportion and $\rho^{*}$ over the century, and neutral ADMs less impacted by the climate scenario in the projections. The composition of community clusters (figure 2) for the five response groups of ADMs are shown bottom right.

Given the sensitivity of biotic interactions to environmental shifts, ADMs allowed us to explore the evolution of ecological associations, to predict potential impacts of climate change on plankton community structure and functions. To conduct this analysis, we considered projections of the IPSL-CM6A-LR RCP 4.5 climate model [47] starting from 2015 and, from 2020 to 2100 with a 10-year interval, resulting in 10 time points. We kept the climate model common grids over these 10 time points, ensuring that the observed feature range covered the feature values (electronic supplementary material, S5). The substantial loss of grids mainly concerned nitrate, for which we have observed concentrations in climate models that are a factor of 10 lower than observed values. By applying this grid filtering, we ensured that the model results are not essentially extrapolated in a statistically biased way by these extremely low nitrate concentrations, since nitrate is a relatively important parameter in establishing biogeographical patterns (figure 2*b*) and ecological relationships are often more complex and non-linear [72–74]. This resulted in coverage of the southern subpolar region, and of mainly coastal regions relatively rich in nitrates, and a region of the west Pacific Ocean (figure 3*a*). According to this climate model, we expect a gradual average rise in temperature in these areas [47,75], coupled with an average decline in nitrate average concentrations throughout the twenty-first century, driven by a strong negative correlation (Pearson = −0.92, *p* = 0.0001). Silicate concentrations are also expected to decrease, though to a lesser extent. Future warming is projected to enhance ocean stratification, reducing the vertical supply of nutrient-rich subsurface waters to the nutrient-depleted surface waters of the euphotic zone [76]. In contrast, salinity does not exhibit significant variations in response to this climate scenario.

We analysed 2667 ADMs that exhibited at least 30% co-presence in cADMs projections for all time points between 2015 and 2100 (electronic supplementary material, S1.3). Among these, 1951 belonged to the subpolar-like cluster, 653 to the widespread-like cluster, 63 to the oligotrophic-like cluster, and only 7 to the tropical-like cluster, which was subsequently excluded from further analysis for its lack of representativeness. To explore the predicted evolution of ecological associations until the end of the century, we examined the evolution of their average percentage of co-presence and $\rho^{*}$ with time. Overall, the average percentage of co-presence tended to decrease (54.0% in 2015 to 51.3% in 2100), primarily driven by the subpolar cluster (56.2% in 2015 to 51.5% in 2100), which was the most represented (figure 3*b*, higher panel). Conversely, associations from the widespread-like clusters showed a slight increase in co-presence proportion (49.0% in 2015 to 51.5% in 2100). No particular trend is observed for the oligotrophic-like cluster (39.8% in 2015 to 39.0% in 2100). Regarding $\rho^{*}$ predictions (figure 3*b*, lower panel; electronic supplementary material, S2), there was a slight overall increase in the oligotrophic-like cluster (from 0.38 to 0.41). In contrast, the widespread-like cluster is predicted to experience a slight reduction in average statistical association strength (from 0.60 to 0.58). These changes, although relatively small and limited to reduced and specific areas of the ocean, nevertheless reflect the utility of ADMs in predicting the potential evolution of community ecology responses to ongoing climate change. When investigating the predicted impact of climate evolution on the overall topological structure of the community, average associations (edge) density is predicted to increase, along with a decrease in average edge betweenness centrality (electronic supplementary material, figure S11), coinciding with relatively significant predicted changes in temperature and nitrates in 2040, 2060 and 2090 (see black arrows on figure 3*a*). Along with the temperature rise, nutrients are likely to play an essential role as a critical driver for the oligotrophic-like cluster, and their decrease may partly explain the overall increase of association statistical strength average. On the other hand, the widespread-like cluster appears to expand its average coverage over the area but experienced a reduction in average association strength. Both clusters suggest potential shifts or expansions of predicted ecological associations. For the subpolar group, rising temperatures likely influence associations predicted to move poleward. Modelling studies in the Southern Ocean have predicted the permanently open ocean zone to experience a poleward shift as a consequence of strengthening wind, and intensified upwelling of deep warm-water masses causing disruptions in nutrient supply [77,78].

Next, to summarize and characterize the temporal responses of co-occurrence associations and ecological association strengths $\rho^{*}$, we computed and compared regression slopes of predicted $\rho^{*}$ and co-presence proportions evolution over time. A higher positive slope indicates an increase in co-occurrence proportion or statistical strength over time, while a negative slope indicates a decrease. To capture associations most impacted by climate change, we defined ranges to classify associations into five response groups (figure 3*c*, §4d(iv)) as follows: (i) 168 (+c)(+r)ADMs experiencing an increase in co-occurrence proportion and associative strength over time, distributed in the widespread-like cluster and oligotrophic-like cluster; (ii) 319 (+c)(−r)ADMs experiencing a decrease in association strength mainly found in the widespread-like cluster; (iii) 1313 (−c)(+r)ADMs experiencing spatial niche reduction but gain in association strength, with the majority belonging to the subpolar-like cluster and a significant proportion in the oligotrophic-like cluster; (iv) 103 (−c)(−r)ADMs decreasing in both metrics and predominantly found in the subpolar-like cluster; and (v) the 1509 ‘neutral’ ADMs, predicted to be not significantly impacted by climate change. The (−c)(−r)ADMs group (*n* = 103) is numerically the smallest, suggesting that associations negatively affected in terms of both co-occurrence and co-abundance are relatively few in the area under consideration. The cADMs representing these associations showed an increased sensitivity to temperature (electronic supplementary material, figure S10). Similarly, the (−c)(+r)ADMs group, mainly composed of subpolar-like cluster associations, displays greater sensitivity to temperature change than other response groups. Thus, temperature sensitivity appears to be a common characteristic among associations predicted to experience reduced co-presence. However, what sets (−c)−r)ADMs apart from (−c)(+r)ADMs is their higher sensitivity to nutrient levels affecting both co-occurrence and co-abundance distributions (electronic supplementary material, figure S10), potentially owing to the higher proportion of associations involving Pelagibacterales (SAR11, Alphaproteobacteria) and Rhodobacterales (Alphaproteobacteria), known to be involved in nitrate metabolism [66,79]. The (+c)(−r)ADMs group mainly involved widespread-like associations predicted to decrease in association strength, which could be the result of these associations expanding their spatial co-presence range, possibly leading to the reduction of their associative strength in a distinct environmental context. Interestingly, this group exhibited relatively low sensitivity to temperature but is predicted to be more impacted by salinity and nutrient concentrations, especially when compared to neutral (−c)ADMs. Finally, (+c)(+r)ADMs (*n* = 168), although small in number, also showed a lower sensitivity to temperature, but higher sensitivity to nutrients and salinity, highlighting the central role of these variables in modulating ecological associations and community structure [40]. Considering the taxonomy of projected associations, (+c)ADMs were enriched in associations involving Alphaproteobacteria (notably Pelagibacterales (SAR11) suspected to expand their distribution poleward [80]) and Gammaproteobacteria, as involving mainly the widespread-like cluster (electronic supplementary material, figure S12). On the other hand, (−c)ADMs are marked by enriched associations implying Chlorophyta (Mamiellales), major contributors to primary production [81], with Arthropods (Marine Hexanauplia B) and prokaryotes, including Cyanobacteria (Synechococcales), Bacteroidota (Flavobacteriales), Alphaproteobacteria (Rhizobiales and Rhodobacterales) or Archaea (Thermoplasmatota) (electronic supplementary material, figure S12). These results highlight that (−c)ADMs and (+c)ADMs represent two distinct major sensitivity community groups where associations losing spatial co-presence are primarily affected by temperature, while nutrients and salinity levels mainly drive those gaining spatial co-presence.

### (d) Predicted impacts of community evolution on marine ecosystem functions

To predict the functional responses of communities to environmental changes, we leveraged the information provided by functional annotations of MAGs (§4a). We identified regions most likely to experience significant shifts in functional potential, focusing on energy metabolism using the Kegg Orthology (KO) database, as it encompasses KO categories involved in the regulation of carbon and nitrogen biogeochemical cycles [82–84]. The biological carbon pump constitutes the biologically driven fixation, export and carbon sequestration from the atmosphere to the ocean [85]. This vital process is mainly mediated by prokaryotic and eukaryotic phytoplankton through photosynthesis [1], but also through diurnal vertical movements of zooplankton as they graze on phytoplankton at the surface and then transport carbon downward by sinking back to the ocean’s depths [86]. While the carbon pump helps buffer climate deregulation, it is threatened by rising ocean acidification and warming of the ocean [87].

**Figure 4.**
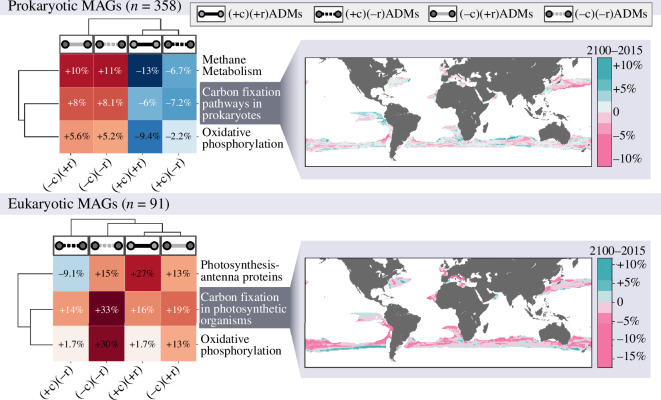
ADMs-based predictions of community functions evolution under climate change. Heatmaps comparing MAGs functional potential related to energy metabolism between responsive and neutral groups (electronic supplementary material, S7). Prokaryotic MAGs involved in (+c)ADMs displayed a predicted depletion of these functions, while the opposite is observed for (−c)ADMs. Almost all eukaryotic MAGs in responsive groups demonstrate an increased potential in energy metabolism functions. A patchy distribution of enrichment or depletion of carbon fixation potential between 2015 and 2100 is depicted for both prokaryotes and eukaryotes. On average, there is a decrease of −0.5 and −2% for prokaryotes and eukaryotes, respectively.

To this end, we considered eukaryotic (*n* = 91) and prokaryotic (*n* = 358) MAGs categorized into the four response groups described above and predicted to exhibit significant distribution changes in ecological associations (figure 3). We compared the sensitive groups with the neutral group to detect enrichment or depletion of the significant functional subcategories of energy metabolism (see electronic supplementary material, S7 for technical details). For prokaryotes, we predicted an enrichment trend in methane metabolism, carbon fixation pathways and oxidative phosphorylation among MAGs included in (−c)(+r)ADMs and (−c)(−r)ADMs predicted to decrease their co-presence proportions in the studied area (figure 4, electronic supplementary material, S7). Conversely, MAGs involved in (+c)(−r)ADMs and (+c)(+r)ADMs are depleted in KOs of the same functional categories. Notably, (−c)(+r)ADMs and (−c)(−r)ADMs are mostly affiliated with the subpolar cluster, which would primarily explain these functional differences. These findings may imply a region-wide decrease in the support of these functions, as enriched MAGs are involved in associations predicted to undergo a loss of co-presence. For eukaryotes in particular, (−c)(−r)ADMs exhibited an enrichment of KOs in carbon fixation in photosynthetic organisms and oxidative phosphorylation functions compared to MAGs in neutral ADMs. In contrast, (+c)(+r)ADMs displayed an enrichment for photosynthesis through antenna proteins, suggesting an overall shift in the actors supporting carbon uptake. This result aligns with other studies predicting alteration in this function, notably in the subpolar region of the Southern Ocean [78], a region mainly contributing to carbon uptake in the ocean [88]. However, these results should be interpreted cautiously, as they include only 91 eukaryotic MAGs.

We projected the predicted biogeography evolution of carbon fixation between prokaryotes and eukaryotes and its predicted evolution between 2015 and 2100 (figure 4 and electronic supplementary material, S7). By comparing carbon-fixation metabolic pathways between 2100 and 2015 over the projected region, ADM projections predict a decrease of −0.5% and −2% for prokaryotes and eukaryotes, respectively. Nonetheless, the biogeography of the evolution of these functions, for both prokaryotes and eukaryotes, is patchy, and we observed spatial heterogeneity in the increase and decrease of carbon-fixation pathways. Collectively, ADM projections have the potential to capture the spatial heterogeneity of community response on a local scale and, at the same time, to draw a global trend for these responses in a changing climate.

## 3. Discussion

Biotic interactions play a crucial role in shaping the biogeography of planktonic communities, ultimately regulating the overall ‘health’ of marine ecosystems [19]. To bridge community ecology and biogeography, and support a better integration of biotic interactions into distribution models, we developed the ADM statistical framework. The ADM framework predicts the biogeography of ecological associations and resulting community structures in space and time. When applied to marine microbial plankton genomes, our findings revealed four major biomes or biogeographical signatures, organized along a latitudinal gradient [89], each characterized by specific ecological associations and community structures as well as differential sensitivities to environmental change. ADM projections performed under a RCP4.5 climate scenario supported differential biogeography and restructuring of ecological associations in response to climate change, potentially impacting plankton community ecological connectivity [90,91]. At the functional level, ADM projections indicated a spatial heterogeneity in the enrichment and depletion of carbon fixation pathways, but with an overall global decrease of approximately −0.5% and −2% in prokaryotes and eukaryotes, respectively, consistent with previous literature reports [14,92,93]. Despite a limited number of observed samples in our study (*n* = 80), we could decipher global scale biogeography of ecological associations for planktonic genomes. By increasing the number and diversity of observed samples, we would capture broader ranges of environmental conditions in different regions and across seasons, leading to more robust and reliable predictions of plankton community structures and their predicted responses to climate change [94]. Biotic-dependent environmental parameters, such as nitrate, shape marine plankton community distributions and interactions [95]. Nevertheless, their accurate integration into most climate models remains limited, as they are significantly influenced by the biotic distributions we seek to predict [96]. This lack of precision in predicting their future concentrations can lead to substantial uncertainties in forecasting the responses of marine ecosystems to climate change. It is, therefore, necessary to further advance the integration of biotic-related environmental parameters into Earth system models [97,98].

In this work, we only performed a naive comparison of SDM and ADM frameworks in predicting co-occurrence and co-abundance distributions in order to introduce a valid conceptual approach to stand alongside the well-known SDM. However, we must emphasize that this may not represent a fair and valid comparison between these frameworks since SDMs are intrinsically designed for species and not associations. This is why, to delve deeper and position ADM in the field of distribution modelling approaches, an extended comparison of ecological association modelling and prediction between ADMs is needed, as well with SDMs integrating indices of biotic dynamics [26], and community distribution modelling approaches, including the so-called jSDMs [34].

Distribution models generally embrace a correlative approach to predict the effects of climate change, by correlating species occurrence data with spatial environmental data [99]. This aligns with the correlative nature of ecological associations within the ADM framework. While ecological associations can serve as statistical proxies for potential biotic interactions, they cannot distinguish direct and indirect interactions, or disentangle true biotic interactions from overlapping environmental niche associations [100]. Moreover, correlative-based models may exhibit poor predictive power when introduced to novel environments, and often provide little insight into the causal mechanisms governing changes in biogeography [101]. To go beyond classical correlations and delve deeper into causality, the integration of metabolic models is a promising avenue [102]. Metabolic cross-feedings, known to mediate planktonic network dynamics [103,104], can provide a mechanistic understanding of the stability and functioning of plankton communities [105]. Combining ADMs with metabolic modelling could provide more robust evidence of effective biotic interactions by predicting metabolic interdependencies between species, and the potential impact of environmental disturbances on these relationships and ecosystem functioning [106]. Beyond its spatial variation, marine plankton is highly dynamic, with temporal fluctuations spanning various temporal scales, encompassing short-term daily [107] and seasonal variations [108] to longer multi-year patterns [101,109]. Recent research has highlighted the need to design models that combine both statistical and dynamical approaches, as conflicts have been observed when both types of models are used independently [101]. In this context, longitudinal datasets will be very valuable to better account for temporal dynamics in marine plankton communities and help to strengthen our confidence in projecting their spatiotemporal distribution [101,110]. Future studies could extend the ADM framework beyond pairwise species interactions by exploring interconnected species modules, modelling them as cohesive units [19]. By modelling and predicting robust plankton communities as a whole, we could unveil the spatial structure of these communities and underlying driving processes [111]. Recent work demonstrated the feasibility of predicting such plankton communities, inferred from global-scale co-occurrence networks, directly from satellite data [112], enabling the long-term spatiotemporal monitoring of plankton communities and their responses to climate change.

ADM represents a significant step towards better comprehending the biogeography and global-scale structure of environmental communities by directly incorporating biotic interaction proxies into distribution modelling. Our exploration of the biogeography of ecological associations and the prediction of their evolution provided valuable insights into the response of plankton communities to climate change. In the future, integrating more resolutive spatiotemporal datasets and climate scenarios with ADM will considerably improve our ability to gain further insights into ecological mechanisms governing marine ecosystems, and improve our capacity to predict their evolution under ongoing climate change.

## 4. Material and methods

### (a) Marine plankton metagenomics data

Prokaryotic and eukaryotic Metagenome-Assembled Genomes (MAGs) abundance tables and taxonomic annotations were downloaded from the *Tara* Oceans databases available at https://www.genoscope.cns.fr/tara/ [54,55]. This resource comprises 2601 MAGs, including 1888 prokaryotic MAGs and 713 eukaryotic MAGs with profiled abundance in 937 samples. Surface samples of three size fractions were selected (0.22–1.6/3, 20–180 and 0.8−5/2000 μm) and the intersection of the shared stations was kept, resulting in 80 samples. Depth and size fraction information characterizing samples were transferred to MAGs (e.g. if MAG1 is observed in samples S1_0.22–1.6/3 and S1_20–180 μm with x and y abundances, respectively, it will be considered as MAGs1_0.22–1.6/3 and MAGs1_20–180 μm with x and y abundances in sample S1). Only MAGs with an occurrence of at least 10 out of 80 samples were retained, resulting in 1887 MAGs (889 MAGs belong to the 0.22–1.6/3 μm size fraction, 249 to 20–180 μm and 747 to 0.8−5/2000 μm). MAGs KO and clusters of orthologous groups (COG) functional annotations were obtained using eggnog-mapper v. 1.0 [113] with the EggNOG v. 5.0 orthology database [114]. To account for the compositional (relative abundance) nature of the metagenomics data, we applied the centred log-ratio (CLR) transformation on the abundance matrix [115] after imputing zeros with a pseudocount following the multiplicative replacement approach proposed by Martín-Fernández [116]. Specifically, the zeros were imputed with the minimum non-zero value multiplied by the factor 0.65 to force an appropriate imputed value below the minimum [117].

### (b) Detection of significant ecological associations

The use of correlations to compositional datasets may lead to the emergence of spurious correlations [118]. On the other hand, proportionality metrics offer meaningful insights in handling compositional data [52]. Precisely, ρ proportionality metric, proposed by Beiyao Zheng [119] and refined by D. Lovell [52], is analogous to correlation since it ranges between −1 and 1. Let n be the number of samples, and $n_{i}$ the *i*th sample for i ranging from 1 to n. Let m be the number of MAGs, and $m_{j}$ the *j*th MAG for j ranging from 1 to m. The global statistical strength $\rho (m_{j},m_{k})$ for each pair of MAGs $\left(m_{j},m_{k}\right)$, where j and k range from 1 to m with j≠k, is outlined in equation (4.1), and was performed using the propr R package [51].


(4.1) $$\rho (m_{j},m_{k})=1-\frac{var(m_{j}-m_{k})}{var(m_{j})+var(m_{k})}$$

When dealing with proportionality, parametric *p* values for each association cannot be calculated [115]. Instead, a false discovery rate (FDR) combined with a bootstrap approach was used to determine negative and positive ρ-thresholds (electronic supplementary material, S4). The FDR was less than 0.01 at −0.35 for the negative ρ-threshold and 0.45 for the positive ρ-threshold, resulting in 103 440 significant pairs out of a possible 1 779 441.

### (c) Inference of rADMs: expansion of global ρ into local statistical contributions α to decompose co-abundance

Associations are statistical models aimed at representing potential biotic interactions. However, the single weight that describes their sign and amplitude is insufficient to capture the spatial variability of biotic interactions. To solve this problem and enable the biogeography of biotic interactions to be modelled, we propose to decompose ecological associations into vectors of local contributions to the overall statistical strength ρ. To decompose ρ, let introduce $x_{ij}^{*}$ be the raw abundance of the *j*th MAG in the *i*th sample. The variance var of the vector CLR-abundances $m_{j}$, where $\bar{m_{j}}$ is the average of $m_{j}$, is defined as:


(4.2) $$var\left(m_{j}\right)=\frac{\sum_{i=1}^{n}{\left(m_{ji}-\bar{m_{j}}\right)}^{2}}{n}.$$

From equation (4.1), we can write:


(4.3) $$\rho \left(m_{j},m_{k}\right)=1-\frac{\sum_{i=1}^{n}{\left(\left(m_{ji}-m_{ki}\right)-\left(\bar{m_{j}}-\bar{m_{k}}\right)\right)}^{2}}{\sum_{i=1}^{n}{\left(m_{ji}-\bar{m_{j}}\right)}^{2}+\sum_{i=1}^{n}{\left(m_{ki}-\bar{m_{k}}\right)}^{2}},$$

(4.4) $$\rho \left(m_{j},m_{k}\right)=1-\frac{\sum_{i=1}^{n}{\left(m_{ji}-m_{ki}\right)}^{2}+2\times \left(m_{ji}-m_{ki}\right)\times \left(\bar{m_{j}-m_{k}}\right)-{\left(\bar{m_{j}-m_{k}}\right)}^{2}}{\sum_{i=1}^{n}{\left(m_{ji}-\bar{m_{j}}\right)}^{2}+\sum_{i=1}^{n}{\left(m_{ki}-\bar{m_{k}}\right)}^{2}}.$$

From this, $\alpha (m_{ji},m_{ki})$, the α score of the *j*th and *k*th MAG at the *i*th sample is defined as:


(4.5) $$\alpha \left(m_{ji},m_{ki}\right)=\frac{-{\left(m_{ji}-m_{ki}\right)}^{2}+2\times \left(m_{ji}-m_{ki}\right)\times \left(\bar{m_{j}-m_{k}}\right)-{\left(\bar{m_{j}-m_{k}}\right)}^{2}}{\sum_{i=1}^{n}{\left(m_{ji}-\bar{m_{j}}\right)}^{2}+\sum_{i=1}^{n}{\left(m_{ki}-\bar{m_{k}}\right)}^{2}}.$$

Hence, ρ can be calculated back from the sum of $\alpha (m_{ji},m_{ki})$ with i going from 1 to n.


(4.6) $$\rho \left(m_{j},m_{k}\right)=1-\sum_{i=1}^{n}\alpha \left(m_{ji},m_{ki}\right),$$

(4.7) $$\sum_{i=1}^{n}\alpha \left(m_{ji},m_{ki}\right)=\alpha \left(m_{j},m_{k}\right)\Rightarrow \rho \left(m_{j},m_{k}\right)=1-\alpha \left(m_{j},m_{k}\right).$$

### (d) Present-day and future projections of association distribution models

#### (i) World Ocean Atlas 18 *in situ* climatology database for present-day projections

The WOA18 database [46] is a resource providing information on the physico-chemical variables in the world’s oceans. Sea surface temperature (SST), salinity, nitrates (NO^3^) and silicates (SiO^4^), known to influence the distribution of planktonic species, were extracted from WOA18. Phosphates were excluded from the analysis owing to known biases in the WOA18 database and high collinearity with nitrates (electronic supplementary material, figure S9). The environmental parameters from WOA18 were mapped to *Tara* samples coordinates and sampling months. Monthly averages of temperature and salinity from 2005 to 2017 and nitrate and silicate averages from the twentieth century were used owing to WOA18 temporal resolution constraints. When an exact grid match was unavailable, we searched within a 2° radius of the sampling location at the closest depth and averaged the values in that area. ADM projections using WOA18 were performed for values at 10 m depth. Grids with feature values between quantiles 2.5 and 97.5 of corresponding observed features were retained, leaving 18′129 grids out of the initial 30′677 grids (electronic supplementary material, S5).

#### (ii) IPSL climate model for future projections

The IPSL-CM6A-LR climate model is an Earth system model (ESM) that integrates various components of the Earth’s climate system [47], including the PISCES biogeochemical model that simulates marine processes like carbon and nutrient cycling. For predictions in the future, we used the IPSL RCP 4.5 scenario, representing medium-level greenhouse gas emissions over the twenty-first century. The same physico-chemical variables as for WOA18 were selected (i.e. SST, salinity, NO^3^ and SiO^4^) whose projections were available from 2015 to 2100 with a one-year step. The Pearson coefficients between the physico-chemical variables of IPSL in 2015 (starting year) and the observed climatology database WOA18 are high (i.e. SST: 0.99, salinity: 0.90, NO^3^: 0.93, SiO4). ADMs IPSL climate model data projections were carried out at 10 m depth. Time points from 2015 and then from 2020 until 2100 with a 10 year interval were used for the analysis, resulting in 10 time points. Common grids to these 10 time points and whose feature values fell within the 2.5 to 97.5 percentiles of the observed feature range were retained, leaving 5655 grids of the initial 32 814 grids between the latitudes of −60 and 60 (electronic supplementary material, S5).

#### (iii) Identifying present-day ADM community clusters

We applied the *k*-means clustering method to the stacked matrix of predicted co-occurrence values to identify ADM-based community ecology clusters from global projections. The optimal number of clusters was determined using the silhouette score from 2 to 15 clusters, with an optimal number of clusters identified for *k* = 4 (electronic supplementary material, figure S4). Edge betweenness topological metrics were computed on reconstructed graphs using the networkx Python library v. 3.1 [120] (https://networkx.org/documentation/stable/reference/algorithms/generated/networkx.algorithms.centrality.edge_betweenness_centrality.html). Betweenness centrality of an edge e is the sum of the fraction of shortest path of all pairs that pass through e. Edge density topological metric is calculated as the ratio of the number of edges per the number of nodes in the graph. The weights of the edges correspond to the reconstructed global $\rho^{*}$, as detailed in electronic supplementary material, S1.2.

#### (iv) Identifying ADM community response groups

We calculated the linear regression slopes of the proportions of co-occurrence and $\rho^{*}$ along the time points and used them to characterize the nature (sign of the slope) and magnitude (value of the slope) of the responses of the ecological associations (using the linregress function of scipy.stats v. 1.11.1 in Python). A slope close to 0 indicates that the climate scenario has no significant impact on the association. Pearson correlation coefficients close to 1, reinforced by $R^{2}$ coefficients close to 1, suggest an overall linear dynamic between time and these response curves, allowing linear regressions to capture response trends. Climate change response groups were identified by setting limits at 10% above and below 0 on the distributions of $\rho^{*}$ and co-occurrence proportions shifts.

## Data Availability

Code repositories for generating ADMs and main and supplementary figures are available at [121]. The data associated with this study are available at [122]. Supplementary material is available online [56].
